# Lithium Chloride Suppresses Colorectal Cancer Cell Survival and Proliferation through ROS/GSK-3****β****/NF-****κ****B Signaling Pathway

**DOI:** 10.1155/2014/241864

**Published:** 2014-06-05

**Authors:** Huili Li, Kun Huang, Xinghua Liu, Jinlin Liu, Xiaoming Lu, Kaixiong Tao, Guobin Wang, Jiliang Wang

**Affiliations:** ^1^Department of Surgery, Union Hospital, Tongji Medical College, Huazhong University of Science and Technology, 430022 Wuhan, China; ^2^Department of Cardiovascular Diseases, Union Hospital, Tongji Medical College, Huazhong University of Science and Technology, 430022 Wuhan, China

## Abstract

Glycogen synthase kinase-3**β** (GSK-3**β**), a serine/threonine protein kinase, has been regarded as a potential therapeutic target for multiple human cancers. In addition, oxidative stress is closely related to all aspects of cancer. We sought to determine the biological function of lithium, one kind of GSK-3**β** inhibitors, in the process of reactive oxygen species (ROS) production in colorectal cancer. In this study, we analyzed the cell apoptosis and proliferation by cell viability, EdU, and flow cytometry assays through administration of LiCl. We used polymerase chain reaction and Western blotting to establish the effect of GSK-3**β** inhibition on the nuclear factor-**κ**B (NF-**κ**B) pathway. Results showed administration of LiCl increased apoptosis and the level of ROS in colorectal cancer cells. Furthermore, the underlying mechanisms could be mediated by the reduction of NF-**κ**B expression and NF-**κ**B-mediated transcription. Taken together, our results demonstrated that therapeutic targeting of ROS/GSK-3**β**/NF-**κ**B pathways may be an effective way for colorectal cancer intervention, although further preclinical and clinical testing are desirable.

## 1. Introduction

Colorectal cancer accounts for approximately 9.7% of all cancers worldwide. It is the third most common cancer and the fourth leading cause of cancer death. The 5-year survival rate for patients with colorectal cancer and metastatic colorectal cancer is less than 60% and 20%, respectively, because of the tumor's resistance to chemotherapy and radiation therapy [1]. Recently, molecular targeting drugs have been suggested for colorectal cancer treatment, but the cost of these drugs is much higher than traditional antitumor drugs. Thus, the identification of novel therapeutic targets and drugs with lower price in colorectal cancer is urgently needed.

Lithium has been an FDA-approved and preferred drug for the treatment of mood disorders for many years, and cumulative evidence has pointed to its potential use as an anticancer agent. Lithium could alter the biochemical properties of a variety of transcription factors and thus exert important physiological or pathophysiological functions in cancer development. There are diverse factors that contribute to colorectal cancer progression and chemoresistance. The transcription factor NF-*κ*B has been shown to be crucial for tumor progression and chemoresistance in colorectal cancer by increasing expression of some target genes such as antiapoptotic Bcl-2 protein and survivin [2]. And previous studies suggest a positive role for GSK-3*β* in the regulation of NF-*κ*B activity [3–6]. It has been demonstrated that lithium is a specific and noncompetitive inhibitor of GSK-3*β* in vitro and in vivo, and consequently it may be an inhibitor of the NF-*κ*B pathway. Although recent studies suggest this drug inhibits cell cycle progression and cell proliferation via upregulating the expression of GSK-3*β* in different cell types [7–10], the role of lithium in the proliferation and survival of colorectal cancer remains elusive.

In this study, for the first time, we present evidence that pharmacological inhibition of GSK-3*β* by lithium treatment can inhibit colon cancer cell line SW480 survival and proliferation in a dose and time-dependent pattern. Moreover, we observed that lithium treatment can induce the accumulation of reactive oxidative species and inhibit the activation of GSK-3*β*, as well as the expression of NF-*κ*B and its target genes Bcl-2 and survivin. All the results demonstrated that lithium could increase the generation of reactive oxygen species (ROS) and lead to decreased cell survival and proliferation via the ROS/GSK-3*β*/NF-*κ*B pathway. Our results suggested that GSK-3*β* could be a novel potential therapeutic target in the treatment of colorectal cancer and lithium should be a novel potential antitumor drug with lower price.

## 2. Materials and Method

### 2.1. Reagents and Cell Culture

Lithium chloride (LiCl, a conventional GSK-3*β* inhibitor) was purchased from Sigma. Human colon cancer cell line SW480 was purchased from the American Type Culture Collection (ATCC, Manassas, VA, USA). Cells were maintained in PRMI 1640 (Gibco, Grand Island, NY, USA) supplemented with 10% fetal bovine serum (Gibco), 100 U/mL penicillin, and 100 *μ*g/mL streptomycin (Invitrogen) and kept in a humidified atmosphere at 37°C with 5% CO_2_ in air.

### 2.2. Cell Viability Assay

To determine the cell viability, cells were plated onto 24-well plates (5 × 10^4^ cells/well). After 24 h incubation with different concentrations (0 mM, 10 mM, 20 mM, 40 mM, and 60 mM) of LiCl and incubation with 40 mM LiCl for different times (6 h, 12 h, 24 h, and 48 h), 0.5 mg/mL 3-(4,5-dimethylthiazole-2-yl)-2,5-diphenyl tetrazolium bromide (MTT, Sigma) was added to the cell suspension for further 4 h, followed by addition of dimethyl sulfoxide (DMSO, Sigma) at 100 *μ*L/well for cell lysis. Then, absorbance was measured at 562 nm. Each assay was carried out in triplicate.

### 2.3. Cell Proliferation Assay

Cell proliferation was determined by using Cell-Light 5-ethynyl-2-deoxyuridine (EdU) DNA Cell Proliferation Kit (RIBOBio. Co., Ltd) [11]. Briefly, cells (1 × 10^5^) were cultured in 24-well plates. After stimulation with different concentrations (0 mM, 10 mM, 20 mM, 40 mM, and 60 mM) of LiCl for 24 h, cells were exposed to 50 *μ*M EdU for 2 h at 37°C. The cells were then fixed in 4% formaldehyde for 30 min at room temperature and permeabilized in 0.5% Triton X-100 for 10 min. Cells were washed with PBS, and each well was incubated with 200 *μ*M 1 × Apollo reaction cocktail for 30 min, followed by 1 × Hoechst 33342 (200 mL per well) for staining nuclei. The stained cells were imaged under a fluorescent microscope (IX71, Olympus).

### 2.4. Determination of Apoptosis

Apoptosis in the colon cancer cell line was measured using the Annexin V-FITC/7-AAD Apoptosis Detection kit (BD Bioscience, CA, USA). After 24 h incubation with different concentrations (0 mM, 10 mM, 20 mM, 40 mM, and 60 mM) of LiCl in 6-well plates at a density of 2 × 10^5^ cells/well, fluorescent intensities were determined by flow cytometry (Becton-Dickinson, CA, USA). Alive cells grouped in the lower left part of the panel, early apoptotic cells grouped in the lower right part of the panel, and late apoptotic cells grouped in the higher right part of the panel. The experiment was repeated at least three times.

### 2.5. Measurements of ROS Generation

Colon cancer cells were cultured in 24-well plates, and after stimulation with different concentrations (0 mM, 10 mM, 20 mM, 40 mM, and 60 mM) of LiCl for 24 h, cells were loaded with the fluorescent dye H2DCF-DA (10 *μ*M, Beyotime Institute of Biotechnology, Haimen, China) for 20 minutes at 37°C. H2DCF-DA fluorescence was detected at excitation and emission wavelengths of 488 nm and 520 nm, respectively. ROS formation was measured using a multiwell fluorescence scanner (EnSpire 2300, Perkin Elmer, USA).

### 2.6. Western Blotting Analysis

Protein extracted from cells stimulated with different concentrations (0 mM, 10 mM, 20 mM, 40 mM, and 60 mM) of LiCl for 24 h was separated on 10% SDS-polyacrylamide electrophoresis gels and transferred to nitrocellulose membranes. Membranes were then blocked with 5% nonfat milk in TBS for 3 hours. After incubation with primary antibodies against GSK-3*β* and its fractions phosphorylated at the serine 9 residue (phospho-GSK-3*β* ^Ser9^) (diluted 1 : 1000; Cell Signaling Technology, Beverly, MA, USA), the tyrosine 216 residue (phospho-GSK-3*β* ^Tyr216^) (diluted 1 : 1000; BD Biosciences), NF-*κ*B p65 (diluted 1 : 1000, R&D), Bcl-2 (diluted 1 : 200, Santa Cruz), survivin (diluted 1 : 200, Santa Cruz), *β*-actin (diluted 1 : 500, Santa Cruz), respectively, in TBS at 4°C overnight, followed by incubation with HRP-conjugated secondary antibody (diluted 1 : 5000) for 3 h. Specific band was detected with chemiluminescence assay (ECL detection reagents, Pierce). The intensity of the *β*-actin band was used as a loading control for comparison between samples.

### 2.7. Real-Time RT-RCR

Total RNA from cultured and stimulated cells (with different concentration of LiCl for 24 h) was isolated using Trizol reagent (Takara Biotechnology) according to manufacturer's instruction. 1 *μ*g of total RNA was reverse transcribed using the PrimeScript RT reagent kit (Takara Biotechnology, Dalian, China). The mRNA levels were determined by real-time RT-PCR with ABI PRISM 7900 sequence detector system (applied biosystem) according to the manufacturer's instructions. GAPDH was used as endogenous control. Relative gene expression level was calculated using the comparative Ct method formula 2^−ΔΔCt^. The sequences of primers for PCR were listed in Supplemental Table 1 (see Supplementary Material available online at http://dx.doi.org/10.1155/2014/241864).

### 2.8. Statistical Analysis

Data are shown as mean ± SEM of at least three independent experiments. The significance of differences was estimated by one-way ANOVA. All statistical analyses were performed with SPSS software (version 11.0, SPSS, Inc.), and statistical significance was set at *P* < 0.05.

## 3. Result

### 3.1. LiCl Decreased Survival of  SW480 Cells

**Figure 1:**
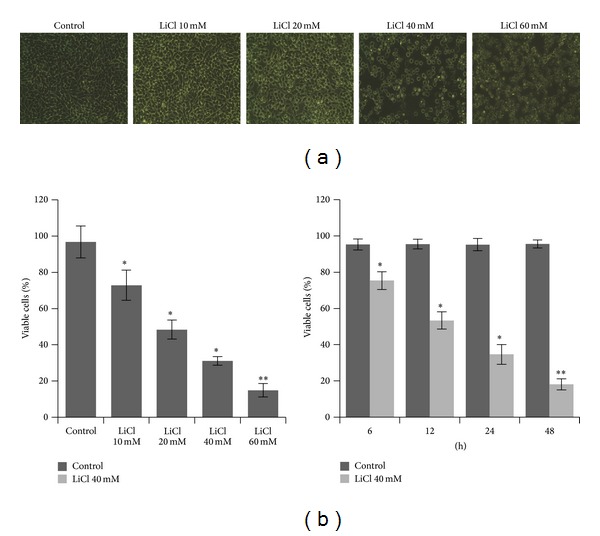
(a) Morphological changes of SW480 cells exposed to different concentrations of LiCl (10 mM, 20 mM, 40 mM, and 60 mM). (b) The percentage of viable SW480 cells. Cells were treated with different concentrations of LiCl (10 mM, 20 mM, 40 mM, and 60 mM) or treated with LiCl for 6, 12, 24, or 48 hours, respectively. Each point is mean ± SEM for at least three individual experiments. Original magnification, ×100.*P < 0.05 and **P < 0.01 versus Control group.

To investigate the role of LiCl in the survival of colon cancer cells, the cell viability was analyzed firstly by light microscopic examination and MTT assay. Light microscopic examination revealed that, compared with the Control group, LiCl caused progressive loss of cell morphology of SW480 cells (Figure 1(a)). Then the cell survival was assessed by MTT method. The results showed that treatment with LiCl caused a gradual reduction in the percentage of viable cells in a dose dependent model. Thereafter the cells were treated with 40 mM LiCl for different times. We found that increase of the incubation time led to a decrease in the percentage of viable cells in time-dependent pattern (Figure 1(b)).

### 3.2. LiCl Suppressed Proliferation of SW480 Cells

**Figure 2:**
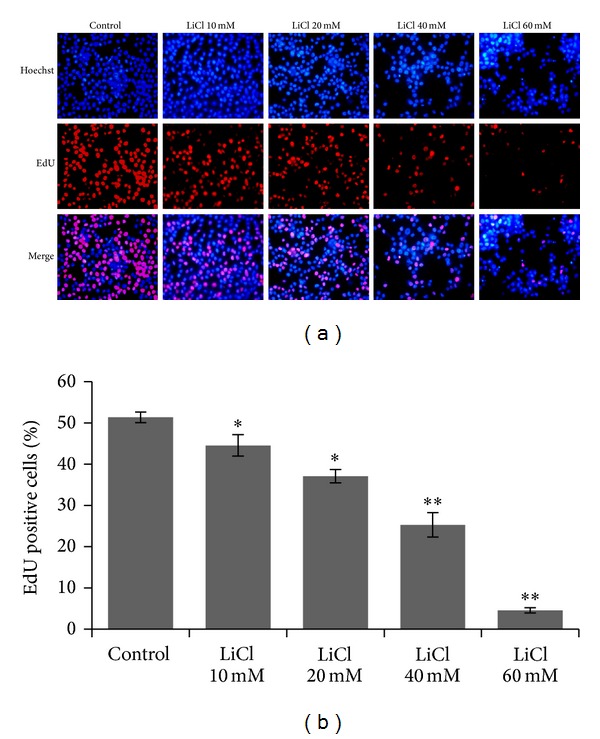
LiCl inhibited proliferation in SW480 cells. Cell proliferation assay was preformed, in which EdU-labeled proliferative cells (red) and Hoechst-stained nuclei (blue) were observed under a fluorescent microscope. Cells were treated with vehicles (PBS) and different concentrations of LiCl (10 mM, 20 mM, 40 mM, and 60 mM), respectively. Data are representative of at least three independent experiments and are expressed as the mean ± SEM. Original magnification, ×100. *P < 0.05 and **P < 0.01 versus Control group.

In order to further characterize the effect of LiCl on the proliferation of colon cancer cells, EdU proliferation assay was also performed. After exposure to different concentration of LiCl for 24 h, the proliferation rate of SW480 significantly decreased from 51.35 ± 1.27% to 44.52 ± 2.59%, 37.09 ± 1.60%, 25.29 ± 2.98%, and 4.58 ± 2.61%, respectively, as shown in Figure 2. These results suggested that LiCl contributed to the reduced proliferation of SW480 cells.

### 3.3. LiCl Induced Apoptosis in SW480 Cells

**Figure 3:**
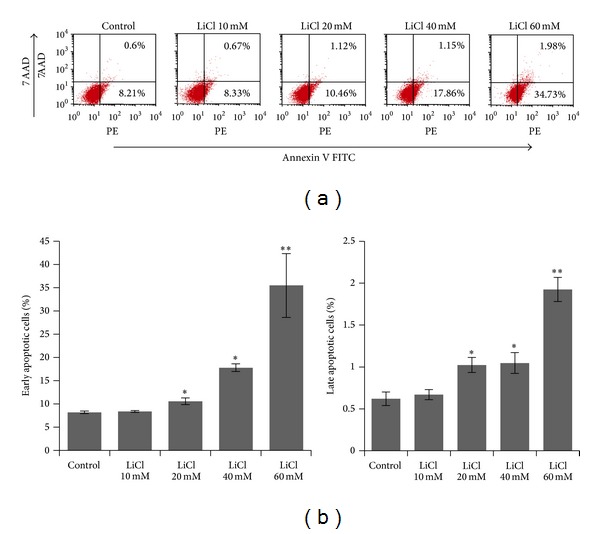
Quantification of early and late apoptosis in SW480 cells treated with different concentrations of LiCl for 24 h was determined by flow cytometry. Representative results were from three independent experiments. Data were shown as mean ± SEM. *P < 0.05 and **P < 0.01 versus Control group.

Since NF-*κ*B is a potential target of GSK-3*β*-dependent cell survival pathway, we detected early apoptotic cells (Annexin V+/7-AAD−) and late apoptotic cells (Annexin V+/7-AAD+) by flow cytometry. Either the early or the late apoptotic cell fractions in the LiCl treated cells were significantly higher than the untreated ones 8.81% (LiCl, 0 mM, 8.21% early, 0.60% late), and the number of apoptotic cells reached 9.00% (LiCl, 10 mM, 8.33% early, 0.67% late), 11.58% (LiCl, 20 mM, 10.46% early, 1.12% late), 19.01% (LiCl, 40 mM, 17.86% early, 1.15% late), and 36.71% (LiCl, 60 mM, 34.73% early, 1.98% late) after 24 h of exposure (Figure 3). It was demonstrated that the number of apoptotic cells dose dependently increased with LiCl treatment. These results confirmed that LiCl treatment led to SW480 cells apoptosis.

### 3.4. LiCl Stimulated ROS Generation

**Figure 4:**
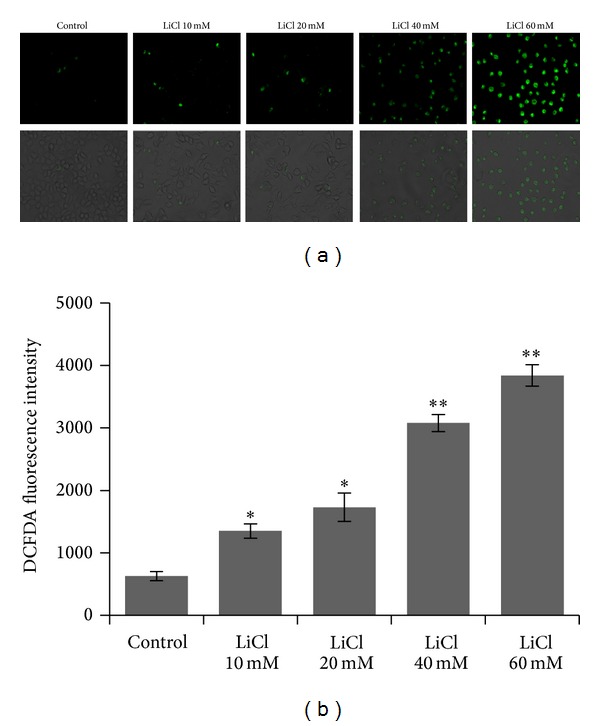
SW480 cells were loaded with a fluorescent probe H2DCF-DA (10 *μ*M) for 20 minutes and observed under fluorescence microscopy. Representative photomicrographs showing ROS within the cytoplasm of cells and merged images showing cell morphology. Fluorescent signals were quantified using a fluorometer at excitation and emission wavelengths of 488 nm and 520 nm, respectively. *P < 0.05 and **P < 0.01 versus Control group. Original magnification, ×200.

To explore the underlying mechanism, we measured the levels of intracellular reactive oxidative species (ROS). In H2DCF-DA loaded SW480 cells treated with different concentration of LiCl (10–60 mM), fluorescence intensity increased in a dose dependent manner, suggesting an increase in the generation of ROS (Figure 4). The results showed that LiCl acted as a prooxidant in colon cancer cells.

### 3.5. Role of GSK-3*β* in LiCl-Mediated ROS Production

**Figure 5:**
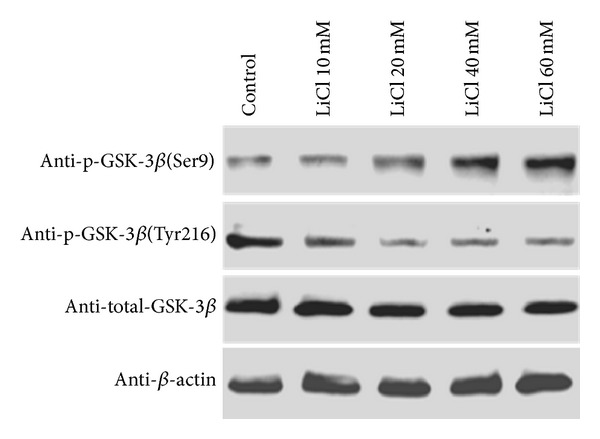
Expression of GSK-3*β* and presence of phosphor-GSK-3*β*(Ser9) (inactive form) and phosphor-GSK-3*β*(Tyr216) (active form) were detected in extracts of SW480 cells treated with LiCl. The amount of protein extract in each sample was monitored by expression of *β*-actin.

To determine whether LiCl might inhibit GSK-3*β* activation under the oxidative damage, immunoblot experiments were performed using an antibody directed against GSK-3*β* and two fractions of phosphor-GSK-3*β* (Ser9 and Tyr216). This allowed us to detect levels of inactive (phosphorylated Ser9) and active (phosphorylated Tyr216) fractions of GSK-3*β*, respectively. When LiCl was added to SW480 cells, it was able to increase the levels of phosphorylation at Ser9, while reducing that at Tyr216 of GSK-3*β* (Figure 5). So it may be reasonable to speculate that GSK-3*β* inhibition was involved in the prooxidant effects of LiCl.

### 3.6. LiCl Inhibited GSK-3*β* Activity and NF-*κ*B Pathway Expression

**Figure 6:**
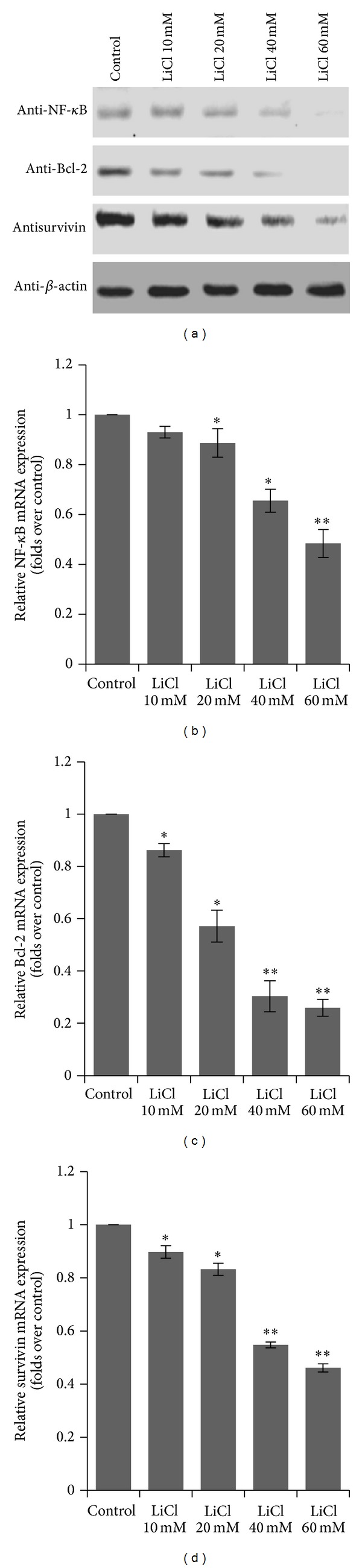
Effect of LiCl on the expression of NF-*κ*B, Bal-2, and survivin protein and mRNA levels. (a) The protein expression of NF-*κ*B, Bal-2, and survivin. (b-c) The mRNA levels of NF-*κ*B, Bal-2, and survivin were analyzed by real-time PCR. The SW480 cells treated with vehicles (PBS) and different concentrations of LiCl (10 mM, 20 mM, 40 mM, and 60 mM), respectively. Data are expressed as mean ± SEM for at least three individual experiments. *P < 0.05 and **P < 0.01 versus Control group.

Multiple factors contribute to colon cancer progression, including activation of NF-*κ*B, whose target genes have an important function in cancer cell survival. As the involvement of GSK-3*β* in the regulation of NF-*κ*B activity is known to be significant for cancer cell growth, we investigated the levels of NF-*κ*B pathway including Bcl-2 and survivin in LiCl treated SW480 cells. Treating with LiCl decreased NF-*κ*B expression in a concentration dependent manner (Figure 6(a)). Then we determined the effect of LiCl treatment on apoptosis-related proteins in SW480 cells. The expression of the NF-*κ*B-regulated genes Bcl-2 and survivin was decreased by treating with LiCl in a concentration dependent manner too. Real-time PCR and immunoblotting had got the same results (Figures 6(b)–6(d)).

## 4. Discussion

The present study showed that LiCl played a critical role in the survival, proliferation, and apoptosis of colorectal cancer cell via the ROS/GSK-3*β*/NF-*κ*B pathway. Our results suggest that targeting GSK-3*β* may be of benefit for the therapeutic activity of anticancer drugs against colorectal tumor.

Oxidative stress is an overproduction of reactive oxygen species that overwhelms the cellular antioxidant capacity. So it seems that increased ROS generation and oxidative stress have the central role in lithium cytotoxic mechanism. In our study, we showed for the first time that LiCl exerted the prooxidant effects in colon cancer cells. Previous studies have demonstrated that administration with lithium for 15 minutes significantly increased intracellular ROS levels in rat hepatocytes [12]. On the contrary, another group showed that, following chronic treatment of neuronal cells with lithium, this ion exhibited obvious antioxidant effects [13]. So the role of lithium as well as the implication of GSK-3*β* in the induction of ROS remains an area of intensive research.

GSK-3 is a pluripotent serine-threonine kinase with numerous intracellular target proteins. GSK-3 isoforms are encoded by distinct genes: GSK-3*α* and GSK-3*β* [14]. GSK-3*β*, known to be a survival factor for cancer, is constitutively activated in colorectal cancer cells. Deregulation of GSK-3*β* has been implicated in tumorigenesis and cancer progression including that of colorectal cancer [15]. LiCl has been shown to induce cell growth arrest, apoptosis, and terminal differentiation in various human malignant tumors by targeting GSK-3*β* [16]. So we used LiCl to inhibit the activity of GSK-3*β* pharmacologically and found the increased generation of ROS, which implied the involvement of ROS in inactivation of GSK-3*β* in the regulation of cancer development.

NF-*κ*B is an important transcription factor involved in growth arrest and apoptosis by regulating the expression of numerous target genes such as Bcl-2 and survivin [17, 18]. Analysis of Bcl-2 and survivin expression levels had been proved to be a useful diagnostic marker and an important source of prognostic information in cancer. NF-*κ*B activity could be boosted by chemotherapeutic stress, leading to tumor chemoresistance. Inactivation of NF-*κ*B could make cancer cells more sensitive to chemotherapy. Here, we found that inhibition of GSK-3*β* suppresses NF-*κ*B-mediated expression of Bcl-2 and survivin. So we provided evidence that GSK-3*β* may serve as a therapeutic target in colorectal cancer.

Colorectal cancer is the malignant tumour occurring in the colorectal lumen, and the colorectal lumen can be connected with the outside through the anus. This makes contact with lump body of colorectal cancer and locoregional therapy through colonoscopy or other possible means. This is the difference on the treatment between colorectal cancer and other kinds of tumors. In this study, we treated colorectal cancer cells with high concentration of lithium chloride, as we considered the convenience of local treatment for colorectal tumors. With the help of colonoscopy, implementation of high drug concentration in local areas of tumors in colorectal lumen is possible, which could make drugs contacting with cancer tumors directly, in order to achieve local targeted therapy and reduce side effects to a minimum. In clinical works, local treatment is one of the important approaches of treatments for colorectal cancer, including local chemotherapy. In patients with possible intestinal obstruction, if the symptoms of intestinal obstruction could be relieved before the surgical operation and supplemented by adjuvant chemotherapy, the success rate for surgical treatment and the prognosis of patients will be greatly improved. In some other investigators' studies, the use of high doses of lithium chloride had been discussed [19, 20]. For example, Suganthi et al. [20] used lithium chloride with the dose of almost 2 times more than ours to observe the effects on tumor cells. This study suggests that the local release of lithium agent around intestinal tumors could promote the apoptosis of the tumor cells and raise the sensitivity of tumors to chemotherapy drugs, which may achieve the goal of lifting intestinal obstruction and avoid side effects caused by systemic application [21–24].

There are several limitations in our work. Firstly, we did not examine the molecular mechanism of GSK-3*β* in the regulation of NF-*κ*B signaling. Secondly, our results were obtained from cell lines, while the situation in the patients with colorectal carcinoma may need more investigation.

In summary, we have demonstrated that inhibition of GSK-3*β* by lithium could induce the production of ROS and suppress the proliferation of colorectal cells by the downregulation of activity of NF-*κ*B and NF-*κ*B-mediated target genes transcription, which may be of benefit for clinical outcome in patients suffering from colon cancer in future.

## 5. Conclusions

Our work has demonstrated a new mechanism of the GSK-3*β* inhibitor lithium; this drug could lead to decreased cell survival and proliferation by the ROS/GSK-3*β*/NF-*κ*B pathway.

## Supplementary Material

The sequences of primers for real time RT-PCR.

## References

[1] Mehrkhani F, Nasiri S, Donboli K, Meysamie A, Hedayat A (2009). Prognostic factors in survival of colorectal cancer patients after surgery. *Colorectal Disease*.

[2] Yang L-Q, Fang D-C, Wang R-Q, Yang S-M (2004). Effect of NF-*κ*B, survivin, Bcl-2 and Caspase 3 on apoptosis of gastric cancer cells induced by tumor necrosis factor related apoptosis inducing ligand. *World Journal of Gastroenterology*.

[3] Coghlan MP, Culbert AA, Cross DA (2000). Selective small molecule inhibitors of glycogen synthase kinase-3 modulate glycogen metabolism and gene transcription. *Chemistry and Biology*.

[4] Hoeflich KP, Luo J, Rubie EA, Tsao M-S, Jin O, Woodgett JR (2000). Requirement for glycogen synthase kinase-3*β* in cell survival and NF-*κ*B activation. *Nature*.

[5] Ougolkov AV, Bone ND, Fernandez-Zapico ME, Kay NE, Billadeau DD (2007). Inhibition of glycogen synthase kinase-3 activity leads to epigenetic silencing of nuclear factor *κ*B target genes and induction of apoptosis in chronic lymphocytic leukemia B cells. *Blood*.

[6] Ougolkov AV, Fernandez-Zapico ME, Savoy DN, Urrutia RA, Billadeau DD (2005). Glycogen synthase kinase-3*β* participates in nuclear factor *κ*B-mediated gene transcription and cell survival in pancreatic cancer cells. *Cancer Research*.

[7] Pardo R, Andreolotti AG, Ramos B, Picatoste F, Claro E (2003). Opposed effects of lithium on the MEK-ERK pathway in neural cells: inhibition in astrocytes and stimulation in neurons by GSK3 independent mechanisms. *Journal of Neurochemistry*.

[8] Stump RJW, Lovicu FJ, Ang SL, Pandey SK, McAvoy JW (2006). Lithium stabilizes the polarized lens epithelial phenotype and inhibits proliferation, migration, and epithelial mesenchymal transition. *Journal of Pathology*.

[9] Sun A, Shanmugam I, Song J, Terranova PF, Thrasher JB, Li B (2007). Lithium suppresses cell proliferation by interrupting E2F-DNA interaction and subsequently reducing S-phase gene expression in prostate cancer. *Prostate*.

[10] Wang J-S, Wang C-L, Wen J-F, Wang Y-J, Hu Y-B, Ren H-Z (2008). Lithium inhibits proliferation of human esophageal cancer cell line Eca-109 by inducing a G2/M cell cycle arrest. *World Journal of Gastroenterology*.

[11] Salic A, Mitchison TJ (2008). A chemical method for fast and sensitive detection of DNA synthesis in vivo. *Proceedings of the National Academy of Sciences of the United States of America*.

[12] Eskandari MR, Fard JK, Hosseini M-J, Pourahmad J (2012). Glutathione mediated reductive activation and mitochondrial dysfunction play key roles in lithium induced oxidative stress and cytotoxicity in liver. *BioMetals*.

[13] Jope RS, Johnson GVW (2004). The glamour and gloom of glycogen synthase kinase-3. *Trends in Biochemical Sciences*.

[14] Doble BW, Woodgett JR (2003). GSK-3: tricks of the trade for a multi-tasking kinase. *Journal of Cell Science*.

[15] de AraÚjo WM, Vidal FCB, de Souza WF, de Freitas Junior JCM, de Souza W, Morgado-Diaz JA (2010). PI3K/Akt and GSK-3*β* prevents in a differential fashion the malignant phenotype of colorectal cancer cells. *Journal of Cancer Research and Clinical Oncology*.

[16] Fu Z-Q, Yang Y, Song J (2010). LiCl attenuates thapsigargin-induced tau hyperphosphorylation by inhibiting GSK-3*β* in vivo and in vitro. *Journal of Alzheimer’s Disease*.

[17] Arbab IA, Looi CY, Abdul AB (2012). Dentatin induces apoptosis in prostate cancer cells via Bcl-2, Bcl-xL, Survivin downregulation, caspase-9, -3/7 activation, and NF-*κ*B inhibition. *Evidence-Based Complementary and Alternative Medicine*.

[18] Cao JP, Niu HY, Wang HJ, Huang XG, Gao DS (2013). NF-kappaB p65/p52 plays a role in GDNF up-regulating Bcl-2 and Bcl-w expression in 6-OHDA-induced apoptosis of MN9D cell. *International Journal of Neuroscience*.

[19] Tang H-R, He Q (2003). Effects of lithium chloride on the proliferation and apoptosis of K562 leukemia cells. *Hunan Yi Ke Da Xue Xue Bao*.

[20] Suganthi M, Sangeetha G, Gayathri G, Ravi Sankar B (2012). Biphasic dose-dependent effect of lithium chloride on survival of human hormone-dependent breast cancer cells (MCF-7). *Biological Trace Element Research*.

[21] Sabancı PA, Ergüven M, Yazıhan N (2014). Sorafenib and lithium chloride combination treatment shows promising synergistic effects in human glioblastoma multiforme cells in vitro but midkine is not implicated. *Neurological Research*.

[22] Adler JT, Hottinger DG, Kunnimalaiyaan M, Chen H (2009). Combination therapy with histone deacetylase inhibitors and lithium chloride: a novel treatment for carcinoid tumors. *Annals of Surgical Oncology*.

[23] Bilir A, Erguven M, Yazihan N, Aktas E, Oktem G, Sabanci A (2010). Enhancement of vinorelbine-induced cytotoxicity and apoptosis by clomipramine and lithium chloride in human neuroblastoma cancer cell line SH-SY5Y. *Journal of Neuro-Oncology*.

[24] Nowicki MO, Dmitrieva N, Stein AM (2008). Lithium inhibits invasion of glioma cells; possible involvement of glycogen synthase kinase-3. *Neuro-Oncology*.

