# Supplementary material for: Lithium Chloride Suppresses Colorectal Cancer Cell Survival and Proliferation through ROS/GSK-3β/NF-κB Signaling Pathway
Source: Oxid Med Cell Longev. 2014 Jun 5;2014:241864. doi: 10.1155/2014/241864 (PMC4070474; doi:10.1155/2014/241864)
Supplement: Supplementary file 1 — The sequences of primers for real time RT-PCR. [file 241864.f1.pdf]

Supplemental Table

Table 1 The sequences of primers for real time RT-PCR

Gene	Primer Sequence (Forward and Reverse)
GAPDH	5'- CCACTCCTCCACCTTTGAC -3'
	5'- ACCCTGTTGCTGTAGCCA -3'
NF-κB	5’- GCAGCACTACTTCTTGACCACC -3’
	5’- TCTGCTCCTGAGCATTGACGTC -3’
Bcl-2	5’- ATCGCCCTGTGGATGACTGAGT -3’
	5’- GCCAGGAGAAATCAAACAGAGGC -3’
survivin	5’- GCCCAGTGTTTCTTCTGCTT -3’
	5’- CCGGACGAATGCTTTTTATG -3’
